# Coronary microvascular function and myocardial fibrosis in women with angina pectoris and no obstructive coronary artery disease: the iPOWER study

**DOI:** 10.1186/s12968-016-0295-5

**Published:** 2016-11-04

**Authors:** Naja Dam Mygind, Marie Mide Michelsen, Adam Pena, Abbas Ali Qayyum, Daria Frestad, Thomas Emil Christensen, Adam Ali Ghotbi, Nynne Dose, Rebekka Faber, Niels Vejlstrup, Philip Hasbak, Andreas Kjaer, Eva Prescott, Jens Kastrup, the steering committee of the iPower study, Ida Gustafsson, Peter Riis Hansen, Henrik Steen Hansen

**Affiliations:** 1 Department of Cardiology, Bispebjerg Hospital, University of Copenhagen, Copenhagen, Denmark; 2 Department of Cardiology, Rigshospitalet, University of Copenhagen, Copenhagen, Denmark; 3 Department of Cardiology, Gentofte Hospital, University of Copenhagen, Copenhagen, Denmark; 4 Department of Cardiology, Hvidovre Hospital, University of Copenhagen, Copenhagen, Denmark; 5 Department of Clinical Physiology, Nuclear Medicine & PET and Cluster for Molecular Imaging, Rigshospitalet and University of Copenhagen, Copenhagen, Denmark

**Keywords:** Microvascular dysfunction, Women, Angina pectoris, T1 mapping, Coronary flow velocity reserve, Cardiovascular magnetic resonance, Doppler echocardiography, Positron emission tomography, Diffuse fibrosis, Extracellular volume

## Abstract

**Background:**

Even in absence of obstructive coronary artery disease women with angina pectoris have a poor prognosis possibly due to coronary microvascular disease. Coronary microvascular disease can be assessed by transthoracic Doppler echocardiography measuring coronary flow velocity reserve (CFVR) and by positron emission tomography measuring myocardial blood flow reserve (MBFR). Diffuse myocardial fibrosis can be assessed by cardiovascular magnetic resonance (CMR) T1 mapping. We hypothesized that coronary microvascular disease is associated with diffuse myocardial fibrosis.

**Methods:**

Women with angina, a clinically indicated coronary angiogram with <50 % stenosis and no diabetes were included. CFVR was measured using dipyridamole (0.84 mg/kg) and MBFR using adenosine (0.84 mg/kg). Focal fibrosis was assessed by 1.5 T CMR late gadolinium enhancement (0.1 mmol/kg) and diffuse myocardial fibrosis by T1 mapping using a modified Look-Locker pulse sequence measuring T1 and extracellular volume fraction (ECV).

**Results:**

CFVR and CMR were performed in 64 women, mean (SD) age 62.5 (8.3) years. MBFR was performed in a subgroup of 54 (84 %) of these women. Mean native T1 was 1023 (86) and ECV (%) was 33.7 (3.5); none had focal fibrosis. Median (IQR) CFVR was 2.3 (1.9; 2.7), 23 (36 %) had CFVR < 2 indicating coronary microvascular disease, and median MBFR was 2.7 (2.2; 3.0) and 19 (35 %) had a MBFR value below 2.5. No significant correlations were found between CFVR and ECV or native T1 (*R* ^*2*^ = 0.02; *p* = 0.27 and *R* ^*2*^ = 0.004; *p* = 0.61, respectively). There were also no correlations between MBFR and ECV or native T1 (*R* ^*2*^ = 0.1; *p* = 0.13 and *R* ^*2*^ = 0.004, *p* = 0.64, respectively). CFVR and MBFR were correlated to hypertension and heart rate.

**Conclusion:**

In women with angina and no obstructive coronary artery disease we found no association between measures of coronary microvascular disease and myocardial fibrosis, suggesting that myocardial ischemia induced by coronary microvascular disease does not elicit myocardial fibrosis in this population. The examined parameters seem to provide independent information about myocardial and coronary disease.

## Background

More than half of women with angina-like chest pain referred for clinical coronary angiography (CAG) have no obstructive coronary artery disease (CAD) [1]. While previously considered a benign condition, recent studies have found this condition to be associated with persistent chest pain, reduced quality of life, repeated angiographies and increased cardiovascular morbidity and mortality [1, 2]. A possible explanation for this discrepancy between CAG findings and symptoms could be ischemia caused by coronary microvascular dysfunction (CMD) which is a strong predictor of cardiovascular prognosis [3–5]. CMD cannot be visualized by standard imaging techniques, but by assessing changes in coronary blood flow or vascular resistance. Although endothelium dependent microvascular function can be assessed, most studies of CMD refer to endothelium independent function assessed during adenosine or dipyridamole stress. Prognostic studies have primarily investigated endothelial-independent measures of CMD by assessment of coronary flow velocity reserve (CFVR) invasively during the CAG or by transthoracic Doppler echocardiography (TTDE) of the left anterior descending artery (LAD) [3, 5–8] or by positron emission tomography (PET) measuring myocardial blood flow reserve (MBFR) [4, 9, 10]. Other methods include contrast perfusion echocardiography [11] and invasive thermo-dilution [12]. TTDE CFVR is the least expensive method - easily accessible, non-invasive, and free from radiation [6]. Furthermore, the method has shown good repeatability [13], as well as agreement with invasively measured CFVR assessed with an intracoronary Doppler wire [14–17]. PET measured CMD has also shown to agree with invasive measured CFVR [18].

Myocardial tissue consists of myocytes, blood vessels and nerves distributed in the extracellular volume (ECV). Cardiovascular magnetic resonance (CMR) is a well-validated method to assess expansion of the myocardial ECV, e.g. as seen with fibrosis [19–21]. Fibrosis of the myocardium is associated with impaired ventricular function, remodelling and stiffness. Localized focal myocardial fibrosis can be observed as late gadolinium enhancement (LGE) after administration of gadolinium [22], but the more recently developed technique called T1 mapping is able to quantify diffuse myocardial fibrosis [23, 24] and has been validated against histological myocardial biopsies [19–21].

The iPOWER study (ImProve diagnOsis and treatment of Women with angina pEctoris and micRovessel disease) aims to investigate diagnostic techniques and prognosis of CMD in women with angina-like chest pain and no obstructive CAD [25]. To date, no study has evaluated whether these women with possible CMD have diffuse myocardial fibrosis. The aim of this sub-study from the iPOWER study was to evaluate the association between CMD assessed by CFVR and MBFR, and presence of diffuse myocardial fibrosis assessed by T1 mapping based on the hypothesis that CMD and consequent myocardial ischemia induce diffuse myocardial fibrosis.

## Methods

### Population and baseline assessment

**Fig. 1:**
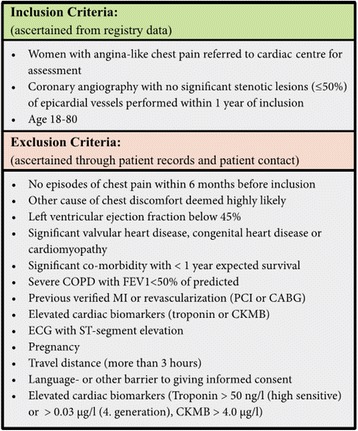
In- and exclusion criteria in the iPOWER study

Women with angina-like chest pain, no significant obstructive CAD assessed by diagnostic invasive CAG with <50 % stenosis of epicardial vessels, and with a successful TTDE CFVR examination were randomly selected for the iPOWER CMR and PET sub-studies [25]. Selection was based on availability of PET and CMR timeslot, participants’ willingness to participate, and minimizing time interval between the examinations. Figure 1 displays the defined in- and exclusion criteria in iPOWER. We included both women referred for stable angina and women hospitalized suspected of unstable angina, since the latter may be first manifestation of stable angina. Further exclusion criteria for the CMR sub-study were diabetes mellitus or contraindication for CMR. Diabetics were excluded as the disease is characterized by microvascular disease in order to avoid it being a confounder. Baseline assessment included clinical and demographic data from interview, charts and the regional CAG database. ECG, blood pressure and heart rate were obtained at rest. Blood samples were analyzed for cholesterol levels (total, low-density lipoprotein [LDL] and high-density lipoprotein [HDL] cholesterol), and triglycerides. Framingham risk score was calculated estimating risk for coronary heart disease over a period of 10 years in women (1 indicating 100 % risk) [26], as well as HeartScore for Denmark (a low risk country) estimating the absolute risk (%) of cardiovascular death within 10 years [27].

Prior to the TTDE, PET and CMR, participants were instructed to be abstinent for 24 h from caffeine or food containing significant amount of methylexanthine (coffee, tea, chocolate, cola and banana) which was confirmed by the study. Medication containing dipyridamole was paused for 48 h, long-lasting nitroglycerines, beta-blockers, ACE-inhibitors, angiotensin-II-antagonist, calcium antagonist, and diuretics for 24 h and short-lasting nitroglycerines 1 h before the examination. This was done in order to avoid an effect on the pharmacological induced hyperemia. Different vasodilators can be used to induce hyperemia, but adenosine and dipyridamole are regarded as equal to achieve peak coronary vasodilation [28, 29] and used interchangeably in clinical practice.

### CFVR measurement and analysis

**Fig. 2:**
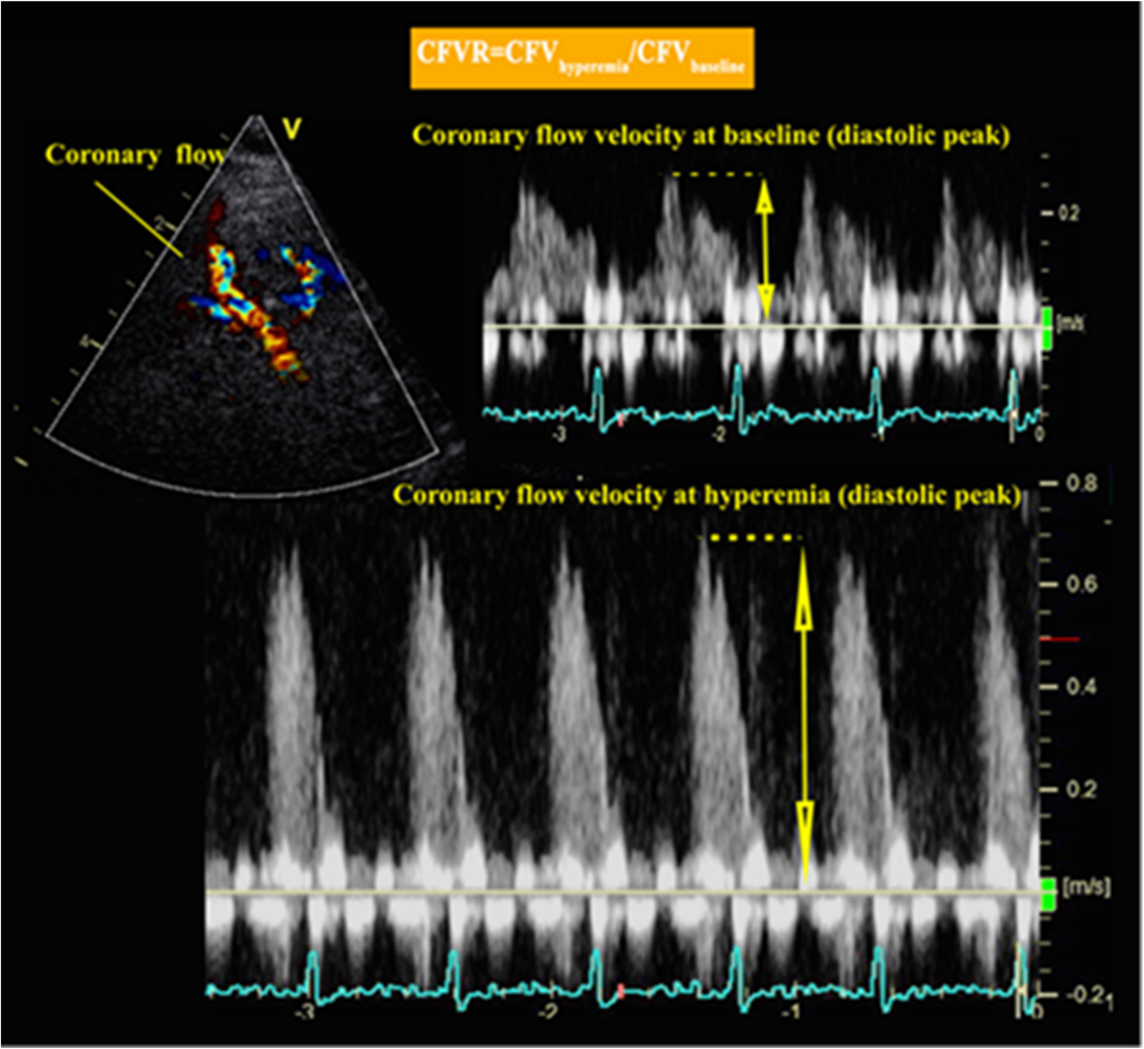
Measurement of coronary flow velocity by transthoracic Doppler echocardiography

At baseline a TTDE of the LAD during rest and high-dose dipyridamole stress (0.84 mg/kg) was performed over 6 min to obtain coronary flow velocities (CFV). The examination was performed using GE Healthcare Vivid E9 cardiovascular ultrasound system (GE Healthcare, Horten, Norway) with a 2.7 - 8 MHz transducer (GE Vivid 6S probe). All examinations were performed by the same 3 experienced echocardiographers in the same settings. CFV was measured by pulsed-wave Doppler as a diastolic laminar flow towards the transducer. Blood pressure and heart rate were measured every 3 min during the examination and after the examination intravenous theophylline (maximum dose 220 mg) was administered to relieve potential side effects of dipyridamole. Diastolic peak flow velocities were analyzed at rest and at peak hyperemia and CFVR was calculated as the ratio between the two (Fig. 2) [30, 31]. In our previous validation study with repeated TTDE CFVR examinations in 10 young, healthy subjects by the same observer we found an intra class correlation coefficient of 0.97 (0.92;1.00) and coefficient of variation (CI) of 7 % for repeat examinations. In a subsample of 50 participants from the iPOWER study, CFVR readings for two analyzers were highly reproducible with a coefficient of variation of 2.7 % [25].

### MBFR measurement and analysis

PET scans were performed using a Siemens Biograph hybrid Computed Tomography (CT)/PET 128-slice scanner (Siemens Healthcare, Knoxville, Tennessee, USA) [10]. Participants underwent serial image acquisition at rest and during adenosine infusion in a single session. For each acquisition, participants received 1.110 MBq (±10 %) Rubidium-82 from a CardioGen-82 strontium-82/Rubidium-82 generator (Bracco Diagnostics Inc., Princeton, New Jersey, USA). Stress Rubidium-82 infusion was initiated 2.5 min after initiation of adenosine infusion. Maximum radiation exposure for the entire examination was 5.2 mSv.

MBF quantification was performed using syngo MBF software (Siemens Healthcare, Knoxville, Tennessee, USA). MBFR was defined as MBF during maximal hyperaemia divided by MBF at rest. MBFR according to the coronary arteries (LAD, right coronary artery [RCA], left circumflex artery [LCX]) was analyzed according to the AHA 17 segment model [32]. Independent observers performed all analyses blinded to results of the TTDE and CMR examination. We did not evaluate the reproducibility of PET MBFR mainly due to ethical reasons as the method exposes participants to radiation. Other groups have evaluated reproducibility of Rubidium-82 PET as well as intra- and interobserver reliability with acceptable agreement [33–35]. We assessed interobserver variability of the MBFR analysis and found a coefficient of variation (CI) of 6.31 % (5.45;7.18).

### CMR

CMR was performed on a Magnetom Avanto 1.5 Tesla scanner (Siemens, Erlangen, Germany). Prior to the scan hematocrit was measured for ECV calculation. A 32 channel chest coil combined with back surface coils was used. Initially scout images were obtained followed by a short-axis cine covering the heart using retrospective ECG gated steady-state free precession (SSFP) cine sequences; field of view 300x300 mm, matrix 192x192, slice thickness 8 mm with 25 number of phases. T1 mapping was performed using the modified look-locker inversion recovery technique (MOLLI) [36]. Before contrast three short-axis MOLLI images were obtained (apical, mid-ventricular and basal). Settings were (5(3)3) referring to 5 acquisition heart beats, followed by 3 recovery heart beats, then a further 3 acquisition heart beats. Field of view 360x360, matrix 218x256, slice thickness 8 mm, flip angle 35°, TR (repetition time) 364.70 ms, TE (echotime) 1.12 ms. TI start was 170 ms with a T1 increment of 80 ms with 2 inversions (TI 250). Gadolinium (Gadovist; Bayer Schering Pharma, Berlin, Germany) (0.1 mmol/kg) was administered followed by 15 ml saline. Post-contrast MOLLI images were obtained 10 min after gadolinium administration with the same settings as pre-contrast except: (4(1)3(1), TR 524.80 ms, TE 1.12 ms and 3 inversions (TI 330) instead of 2.

LGE images were acquired as breath-hold ECG gated, inversion recovery fast gradient-echo images after the T1 post-contrast session. Initially an inversion time (TI) scout was performed to find the best inversion time to null the myocardium. Images were acquired to cover the whole length of the left ventricle; slice thickness 8 mm, TE 3.38 ms; TR 848.00 ms; flip angle 25°; field of view 340x340, matrix 192x192, GRAPPA acceleration factor 2.

### CMR analysis

CMR analysis was performed using commercial available software CVI42 version 5.1.1 (Circle Cardiovascular Imaging Inc., Calgary, Canada) blinded to results of the TTDE and PET examination. All CMR analyses were performed by the same reader, trained by an expert CMR physician. The reader and expert performed double readings until the reader could reproduce the expert satisfactory. The coefficients of variation between the reader and an expert CMR physician were 6.0 % (0.1; 12.0) for LVEF, 2.5 % (0.1; 4.9) for native T1 and 4.4 % (0.1; 8.7) for postcontrast T1 times. Furthermore intra-observer variability was 5.9 % (0.1;11.7) for LVEF, 1.7 % (0.1;3.3) for native T1 and 6.3 (0.1; 12.0) for postcontrast T1. Another group performed repeated MOLLI T1 mapping in 15 healthy volunteers. Mean difference (SD) for repeated examinations were 14.4 ms (34.7) for native T1 and 4.5 ms (15.4) for post-contrast T1. Both intra- and interobserver agreement (native T1) were high, mean difference (SD) 2.6 ms (6.7) and 1.1 ms (8.9), respectively [37].”

Left ventricle mass and volumes were assessed by manually tracing the epi- and endocardial borders of the short-axis cine images and stroke volume and left ventricular ejection fraction calculated (LVEF) [38]. Left ventricular hypertrophy (LVH) was defined as LVH > 61 g/m2 [39]. Presence of LGE was assessed visually.

T1 times images were analyzed to construct a T1 map. Endo- and epicardial borders were drawn on all images divided on 3 slices pre- and post-contrast. The recovery curve was generated and dicom maps created by the software. For the T1 map analysis the CVI42 generated maps were used. Contours of the endo- and epicardial borders were drawn on the 3 slices using a 20 % endo- and epicardial offset and 6 segments per slice. For ECV analysis a region of interest was drawn in the blood pool avoiding the papillary muscles and the anterior and inferior insertion of the right ventricle was marked as reference points.

ECV was calculated by the equation [21]:

$$ \mathrm{E}\mathrm{C}\mathrm{V} = \uplambda *\ \left(1\hbox{-} \mathrm{hematocrit}\right);\kern0.5em \uplambda = \Delta \mathrm{R}1\ \left(\mathrm{myocardium}\right)\ /\kern0.5em \Delta \mathrm{R}1\ \left(\mathrm{blood}\ \mathrm{pool}\right);\kern0.5em \Delta \mathrm{R}1 = \mathrm{R}1\ \left(\mathrm{post}\hbox{-} \mathrm{contrast}\right)\ \hbox{--}\ \mathrm{R}1\ \left(\mathrm{native}\right);\kern0.5em \mathrm{R}1 = 1/\mathrm{T}1 $$

(Fig. 3)

**Fig. 3:**
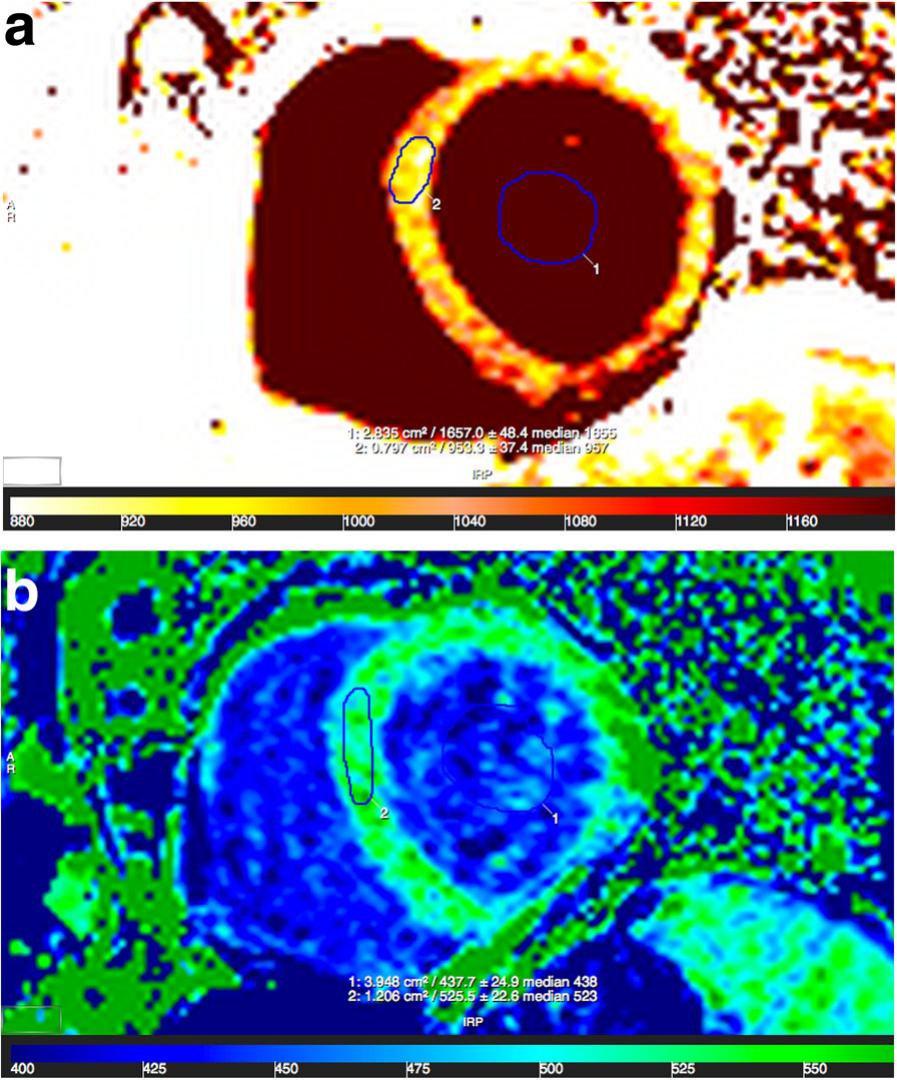
Analysis of T1 mapping CMR images pre- and postcontrast. Legend: Native T1 (**a.**) and postcontrast T1 (**b.**) map. Regions of interests drawn in the myocardium and in the blood pool giving T1 values. The extracellular volume fraction (ECV) was calculated using the T1 times and the hematocrit using the equation: ECV = λ * (1-hematocrit); λ = ΔR1 (myocardium) / ΔR1 (blood pool); ΔR1 = R1 (post-contrast) – R1 (native); R1 = 1/T1

T1 values (native and post-contrast) and ECV was given globally, for each slice (basal, mid-ventricular and apical) and according to the coronary arteries (LAD, RCA, LCX) segments calculated according to the AHA 17-segment model (true apex not imaged) [32]. If a segment was missing due to slices being placed with error during the scan, the artery segment was calculated with a segment less (maximum 2 missing segments per participant).

### Statistical analyses

Continuous variables with a Gaussian distribution are expressed as mean ± standard deviation (SD). Median, interquartile range (IQR) is used for variables with a non-Gaussian distribution. Count in % is used for categorical variables. Distribution was assessed visually. The coefficient of variation between CMR readers was calculated by dividing SD_dif_ with the mean value and expressed as a percentage. The 95 % CI for the estimated coefficient of variation was calculated according to the equation: (SD_dif_ ± t · SE(SD_dif_)/mean) · 100 %.

Difference in T1 variables (native, post-contrast and ECV) according to slice (apical, mid-ventricular and basal) and artery territory (LAD, RCA and LCX) was tested by one-way ANOVA.

Correlations were assessed by Pearson’s correlation coefficient. Logarithm transformed values was used in case of non-Gaussian distribution of one variable. In case both variables were skewed Spearman correlation coefficient was used.

Participants were stratified according to tertiles of native T1 and ECV and into three groups using pre-defined CMD defining cut-off points for CFVR and MBFR to test for differences between groups. Trend-tests by logistic or linear regression analysis were used to evaluate the distribution of variables. Dependent variables with skewed distribution were transformed with the natural logarithm.

To explore predictors of native T1, ECV, CFVR and MBFR multivariable linear regression analyses were performed. All potential explanatory variables with an a priori defined hypothesis were tested in a prioritized order as determinants. Outcome variables with a non-Gaussian distribution were logarithmically transformed using the natural logarithm.

Statistical analysis was performed using SAS Enterprise Guide 7.1, windowed for SAS version 9.4 (SAS institute Inc., Cary, North Carolina; USA). A two-sided *p*-value below 0.05 was considered statistically significant.

## Results

### Study population

From the iPOWER population with a successful CFVR measurement 79 participants were recruited for the CMR study. Two participants could not complete the CMR scan due to claustrophobia and 13 were scanned with different software, making ECV calculation impossible and comparisons unreliable. Thus 64 had a complete CMR for T1 mapping. Mean age (SD) was 62.5 (8.3) years, 70.3 % had stable angina pectoris as indication for the clinical CAG and 45.3 % had non-obstructive atherosclerosis. The duration of angina varied; 38 % had had their angina symptoms under a year and 34 % for more than 3 years. The overall burden of cardiovascular risk factors was relatively high (Table 1). Mean (SD) score in the European Society of Cardiology (ESC)’s HeartScore was 1.4 (1.2) and in the Framingham risk score the mean score was 0.093 (0.05). Median time-interval (IQR) between the TTDE and the CMR was 112 (59; 237) days.

**Table 1:** Demographics, Medical History, Biochemistry and CMR values for the CFVR population and the part also examined by PET (MBFR population)

	CFVR population, *n* = 64	MBFR population, *n* = 54
Age, mean (SD)	62.5 (8.3)	62.0 (7.5)
Hypertension, n (%)	38 (59.4)	29 (53.7)
Hyperlipidaemia, n (%)	42 (65.6)	34 (63)
Family history of CAD, n (%)	36 (57.1)	30 (57)
Smoking (current), n (%)	15 (23)	12 (22)
Smoking (previous + current), n (%)	39 (61)	34 (63)
Pack years (20 cig./day) · year)^a^, median (IQR)	27 (7;35)	29 (8;35)
Stable angina pectoris, n (%)	45 (70.3)	39 (72)
Postmenopausal status, n (%)	56 (89)	48 (89)
Comorbidity, n (%)	38 (60)	31 (59)
ESC’s HeartScore (% risk)^b^, mean (SD)	1.4 (1.2)	1.3 (1.3)
Framingham risk score^c^, mean (SD)	0.093 (0.05)	0.094 (0.06)
Biochemistry		
Total-cholesterol (mmol/l), mean (SD)	4.8 (1.0)	4.9 (1.1)
LDL cholesterol (mmol/l), mean (SD)	2.7 (1.0)	2.8 (1.0)
HDL cholesterol (mmol/l), mean (SD)	1.6 (0.5)	1.6 (0.5)
Non-HDL cholesterol (mmol/l), mean (SD)	3.2 (1.0)	3.3 (1.0)
Hematocrit, mean (SD)	40.5 (2.9)	40.7 (2.7)
Clinical Assessment		
Body mass index (kg/m^2^), median (IQR)	23.9 (21.9;28.3)	23.7 (22.0;27.9)
Body mass index (kg/m^2^) > 25, n (%)	26 (41)	22 (40)
Abdominal circumference (cm), mean (SD)	93.5 (12.4)	93 (12.5)
Systolic blood pressure (mmHg), mean (SD)	148.1 (25.7)	146.5 (25.8)
Diastolic blood pressure (mmHg), mean (SD)	85.5 (15.8)	85.6 (16.5)
Heart rate at rest (bpm), mean (SD)	64.8 (10.3)	64.2 (11.1)
Atherosclerosis at CAG, n (%)	29 (45)	24 (44)
Cardiac Magnetic Resonance (global values)		
Left ventricular ejection fraction (%), mean (SD)	59.6 (5.9)	59.7 (5.9)
End systolic volume (ml), mean (SD)	60.6 (17.2)	61.1 (17.4)
End diastolic volume (ml), mean (SD)	148.5 (27.0)	150.3 (27.8)
Left ventricular mass index (g/m^2^), mean (SD)	48.38 (7.6)	49.1 /7.8)
Left ventricular hypertrophy, n (%)	5 (8)	5 (10)
Cardiac output, mean (SD)	5.7 (1.1)	5.7 (1.2)
Stroke volume, mean (SD)	87.9 (15.3)	89.3 (16.1)
Myocardial mass (diastole), mean (SD)	87.2 (17.8)	88.2 (18.8)
Myocardial mass (systole), mean (SD)	92.6 (20.7)	92.9 (21.5)
Medication		
Beta Blockers, n (%)	23 (36)	19 (35)
Acetylsalicylic acid, n (%)	35 (54.7)	29 (54)
Statin, n (%)	35 (54.7)	28 (52)
Calcium antagonists, n (%)	17 (27)	13 (25)
Angiotensin conv. enzyme inhibitor, n (%)	8 (13)	7 (13)
Angiotensin receptor blockers, n (%)	11 (18)	6 (11)

^a^Only including previous and current smokers. ^b^Estimates absolute risk (%) for cardiovascular death within 10 years. ^c^Estimates risk for coronary heart disease over a period of 10 years (1 = 100 % risk). *CFVR* coronary flow velocity reserve, *MBFR* myocardial blood flow reserve, *IQR* interquartile range, *SD* standard deviation, *CAD* coronary artery disease, *LDL* low-density-lipoprotein, *HDL* high-density-lipoprotein, *non-HDL* non-high-density-lipoprotein cholesterol, *ESC* European Society of Cardiology

Of the 64 participants included and in examined in the iPOWER CMR sub-study 54 (84 %) also had a PET scan performed measuring MBFR. Mean age was 62 (7.5) years and prevalence of cardiovascular risk factors was similar to the total study population (Table 1). Median time-interval between the PET and the CMR was 97 (37; 225) days.

### Measures of diffuse myocardial fibrosis and cardiovascular risk factors

On a global level mean native T1 was 1023 (86) ms, post-contrast T1 463 [33] ms and ECV (%) was 33.7 (3.5). Native and post-contrast T1 times increased significantly from apex to base and ECV was significantly higher towards the apical slice. Native T1 times varied according to coronary artery territory with the highest value in the RCA territory, where ECV also was highest (Table 2). As expected, native T1 and ECV were associated (*p* < 0.001). When stratifying participants in tertiles according to native T1 and ECV no variables were associated with both CMR derived measures of fibrosis: low ECV was associated with more atherosclerosis on the CAG and low native T1 was associated with more hypertension. Native T1 increased with higher resting heart rate (Table 3). In multivariable linear regression analysis none of the examined variables were able to predict ECV and native T1 (data not shown).

**Table 2:** T1 mapping values according to slice and coronary artery territory

	Ventricular Slice				Coronary Artery Territory			
	Basal	Mid	Apical	*p*-value*	LAD	RCA	LCX	*p*-value*
T1 native (ms)	1043 (38)	1016 (57)	976 (117)	<0.0001	992 (48)	1023 (39)	955 (82)	<0.0001
T1 postcontrast (ms)	483 (35)	469 (38)	439 (35)	<0.0001	464 (37)	459 (64)	477 (40)	0.10
ECV (%)	32.8 (3.3)	33.1 (4.0)	34.6 (5.0)	0.04	32.0 (3.0)	32.1 (3.5)	30.2 (4.0)	0.001

*Differences between groups were tested by one-way ANOVA

*ECV* extracellular volume, *LAD* left anterior descending artery, *RCA* right coronary artery, *LCX* left circumflex artery

**Table 3:** Variables according to measures of diffuse myocardial fibrosis

	Extracellular Volume Fraction (%)				Native T1 (ms)			
	<32.3	32.3-34.5	>34.5	*p*-value*	<996	996-1032	T > 1032	*p*-value*
	(*n* = 22)	(*n* = 21)	(*n* = 21)		(*n* = 22)	(*n* = 21)	(*n* = 21)	
T1 native (ms)/ECV (%), mean (SD)	996 (49)	1010 (37)	1065 (129)	<0.001^**^	32.5 (3.2)	33.04 (1.8)	35.6 (4.9)	<0.001^**^
CFVR, median (IQR)	2.2 (1.9;2.6)	2.6 (2.1;2.9)	2.1 (1.7;2.6)	0.27^**^	2.2 (1.7;2.6)	2.5 (2.0;2.9)	2.2 (1.8;2.6)	0.61^**^
MBFR^a^, median (IQR)	2.7 (2.1;2.9)	2.7 (2.2;3.3)	2.7 (2.3;3.0)	0.13^**^	2.6 (2.0;2.9)	2.8 (2.6;3.2)	2.5 (2.1;2.7)	0.64^**^
Age (years), mean (SD)	63.5 (7.9)	60.2 (7.8)	63.6 (9.1)	0.31	64.3 (8.2)	61.5 (8.0)	61.5 (8.8)	0.45
BMI (kg/m^2^), median (IQR)	25 (23;29)	23 (22;26)	23 (22;29)	0.31	24 (22;28)	24 (22;26)	25 (22;29)	0.86
Hypertension, n (%)	15 (68)	12 (57)	11 (52)	0.56	18 (82)	7 (33)	13 (62)	0.01
Smoking (current), n (%)	3 (14)	6 (29)	6 (29)	0.42	4 (18)	3 (14)	8 (38)	0.16
Ever smoked, n (%)	17 (77)	11 (52)	11 (52)	0.17	13 (59)	12 (57)	14 (67)	0.80
Atherosclerosis on CAG, n (%)	16 (73)	6 (29)	7 (33)	0.01	11 (50)	7 (33)	11 (52)	0.41
Non-HDL cholesterol, mean (SD)	3.3 (1)	3.2 (0.9)	3.2 (1.2)	0.84	3.0 (1.1)	3.5 (1.0)	3.3 (0.7)	0.24
Systolic BP (mmHg), mean (SD)	152 (21)	146 (27)	147 (29)	0.71	146 (22)	146 (26)	152 (30)	0.67
Resting HR (bpm), mean (SD)	65 (9.5)	63 (11.8)	67 (9.6)	0.53	65 (11.2)	60 (11.3)	69 (6.5)	0.04
Ejection fraction (%), mean (SD)	61 (5.2)	60 (6.7)	59 (5.9)	0.46	60.9 (4.9)	59.4 (5.7)	58.7 (7.1)	0.48
LV mass index (g/m^2^), mean (SD)	47.9 (6.5)	48.4 (6.6)	48.8 (9.7)	0.94	46.6 (5.5)	50.3 (8.7)	48.4 (8.3)	0.31
LV hypertrophy, n (%)	1 (5)	1 (5)	3 (14)	0.44	0 (0)	3 (14)	2 (10)	0.84
ESC HeartScore (% risk)^b^, mean (SD)	1.64 (1.3)	1.00 (1.2)	1.4 (1.1)	0.23	1.5 (1.1)	1.05 (1.3)	1.5 (1.2)	0.37
Framingham risk score^c^, mean (SD)	0.1 (0.06)	0.08 (0.04)	0.1 (0.06)	0.35	0.09 (0.05)	0.1 (0.06)	0.09 (0.06)	0.90
Beta blockers, n (%)	9 (41)	9 (43)	5 (24)	0.38	10 (45)	8 (38)	5 (24)	0.34
Acetylsalicylic acid, n (%)	13 (59)	12 (57)	10 (48)	0.73	13 (59)	10 (48)	12 (57)	0.73
Statin, n (%)	15 (68)	13 (62)	7 (33)	0.06	15 (68)	10 (48)	10 (48)	0.30
Calcium antagonists, n (%)	7 (32)	6 (29)	4 (19)	0.61	8 (36)	3 (14)	6 (29)	0.31
ACE inhibitor, n (%)	2 (9)	3 (14)	3 (14)	0.82	5 (23)	1 (5)	2 (10)	0.24
Ang.Rec. Blockers, n (%)	3 (14)	3 (14)	5 (25)	0.65	4 (18)	2 (10)	5 (24)	0.52

^*^Difference between groups was tested by trend-test: multiple regression for continuous variables & logistic regression for categorical outcome variables. Log transformed values for the outcome variable was used for the skewed variables. ^**^ *P*-value obtained from Pearson’s correlation coefficient.^a^Participants in the 3 MBFR groups were 18, 19 and 20. ^b^Estimates absolute risk (%) for cardiovascular death within 10 years. ^c^Estimates risk for coronary heart disease over a period of 10 years (1 = 100 % risk)

*ECV* extracellular volume fraction, *CFVR* coronary flow velocity reserve, *MBFR* myocardial blood flow reserve, *IQR* interquartile range, *SD* standard deviation, *BMI* body mass index, *CAG* coronary angiography, *non-HDL*, Non-high-density-lipoprotein cholesterol, *HR* heart rate, *BP* blood pressure, *LV* left ventricle, *ESC* European Society of Cardiology, *ACE* Angiotensin converting enzyme, *Ang.Rec.*, Angiotensin receptor

### Measures of coronary microvascular dysfunction and cardiovascular risk factors

Median CFVR was 2.3 (1.9;2.7) and 23 (36 %) had CFVR below the cut-off of 2. Median MBFR was 2.7 (2.2;3.0) and 19 (35 %) had a MBFR value below 2.5. The two measures of CMD were only weakly correlated (*p* = 0.01, *R* ^*2*^ = 0.132). When stratifying participants according to CMD defining cut-off points for CFVR and MBFR, impaired CFVR was associated with smoking and both CFVR and MBFR were associated with presence of hypertension and a higher resting heart rate (Table 4), as also found in multivariable regression analysis (data not shown).

**Table 4:** Measures of CMD and cardiovascular risk factors

	Coronary Flow Velocity Reserve				Myocardial Blood Flow Reserve			
	<2	2-2.5	>2.5	*p*-value*	<2	2-2.5	>2.5	*p*-value*
	(*n* = 23)	(*n* = 16)	(*n* = 25)		(*n* = 6)	(*n* = 13)	(*n* = 35)	
MBFR/CFVR, median (IQR)	2.2 (2.0;2.7)	2.7 (2.4;2.8)	2.9 (2.5;3.2)	0.01^**^	2.4 (1.9;2.7)	2.0 (1.7;2.3)	2.6 (2.2;2.9)	0.01^**^
T1 native (ms), mean (SD)	1046 (123)	1005 (123)	1014 (51)	0.61^**^	985 (34)	1053 (159)	1013 (55)	0.64^**^
ECV (%), mean (SD)	34.5 (4.5)	32.6 (2.2)	33.7 (2.9)	0.27^**^	31.2 (2.7)	34.2 (3.9)	33.6 (2.6)	0.13^**^
Age (years), mean (SD)	64.0 (10.3)	61.2 (6.4)	61.8 (7.49	0.52	60.2 (7.5)	63.1 (8.6)	61.9 (7.2)	0.74
BMI (kg/m^2^), median (IQR)	24 (22;27)	23 (21;28)	25 (23;29)	0.42	26 (24;29)	23 (22;24)	25 (22;29)	0.46
Hypertension, n (%)	20 (89)	10 (63)	8 (32)	0.002	5 (83)	10 (77)	14 (40)	0.03
Smoking (current), n (%)	6 (26)	4 (25)	5 (20)	0.87	3 (50)	4 (31)	5 (14)	0.13
Ever smoked, n (%)	14 (61)	14 (88)	11 (44)	0.04	5 (83)	10 (77)	19 (54)	0.22
Atherosclerosis on CAG, n (%)	12 (52)	10 (63)	7 (28)	0.08	5 (83)	5 (39)	14 (40)	0.20
Non-HDL cholesterol, mean (SD)	3.2 (1.0)	3.0 (0.9)	3.5 (1.0)	0.36	3.0 (1.2)	3.5 (0.8)	3.2 (1.1)	0.59
Systolic BP (mmHg), mean (SD)	142 (24)	156 (22)	149 (28)	0.28	151 (18)	141 (30)	148 (26)	0.68
Resting HR (bpm), mean (SD)	68 (10)	68 (12)	60 (9)	0.02	68 (15)	70 (9)	61 (104)	0.05
Ejection fraction (%), mean (SD)	58.6 (6.7)	61.7 (4.2)	59.4 (6.0)	0.30	57.6 (10.0)	58.6 (6.1)	60.6 (4.9)	0.39
LV mass index (g/m^2^), mean (SD)	45.8 (6.0)	49.7 (8.2)	50.1 (8.2)	0.12	49.4 (2.3)	47.3 (9.8)	49.9 (7.7)	0.61
LV hypertrophy, n (%)	0 (0)	2 (15)	3 (12)	0.96	0 (0)	1 (8)	4 (11)	0.90
ESC HeartScore (% risk)^a^, mean (SD)	1.3 (1.0)	1.4 (1.2)	1.3 (1.5)	0.91	1.2 (0.8)	1.6 (1.4)	1.2 (1.2)	0.59
Framingham risk score^b^, mean (SD)	0.09 (0.04)	0.1 (0.06)	0.09 (0.06)	0.61	0.1 (0.1)	0.08 (0.03)	0.09 (0.05)	0.67
Beta blockers, n (%)	7 (30)	6 (38)	10 (40)	0.78	1 (17)	5 (38)	13 (37)	0.62
Acetylsalicylic acid, n (%)	14 (61)	7 (44)	14 (56)	0.57	6 (100)	6 (46)	17 (49)	0.10
Statin, n (%)	15 (65)	9 (56)	11 (44)	0.34	6 (100)	5 (38)	17 (48.6)	0.82
Calcium antagonists, n (%)	8 (35)	3 (19)	6 (25)	0.53	3 (50)	3 (23)	7 (21)	0.34
ACE inhibitor, n (%)	2 (9)	4 (25)	2 (8)	0.26	1 (17)	2 (15)	4 (12)	0.92
Ang. Rec. Blockers, n (%)	7 (30)	2 (13)	2 (8)	0.14	0 (0)	2 (15)	4 (12)	0.95

^*^Difference between groups was tested by trend test: multiple regression for continuous variables & logistic regression for categorical outcome variables. Log transformed values for the outcome variable was used for the skewed variables. ^**^ *P*-value obtained from Pearson’s correlation coefficient. ^a^Estimates absolute risk (%) for cardiovascular death within 10 years. ^b^Estimates risk for coronary heart disease over a period of 10 years (1 = 100 % risk)

*ECV* extracellular volume fraction, *CFVR* coronary flow velocity reserve, *MBFR* myocardial blood flow reserve, *IQR* interquartile range, *SD* standard deviation, *BMI* body mass index, *CAG* coronary angiography, *non-HDL* Non-high-density-lipoprotein cholesterol, *HR* heart rate, *BP* blood pressure, *LV* left ventricle, *ESC* European Society of Cardiology, *ACE* Angiotensin converting enzyme, *Ang.Rec.* angiotensin receptor

### Measures of CMD and presence of diffuse myocardial fibrosis

**Fig. 4:**
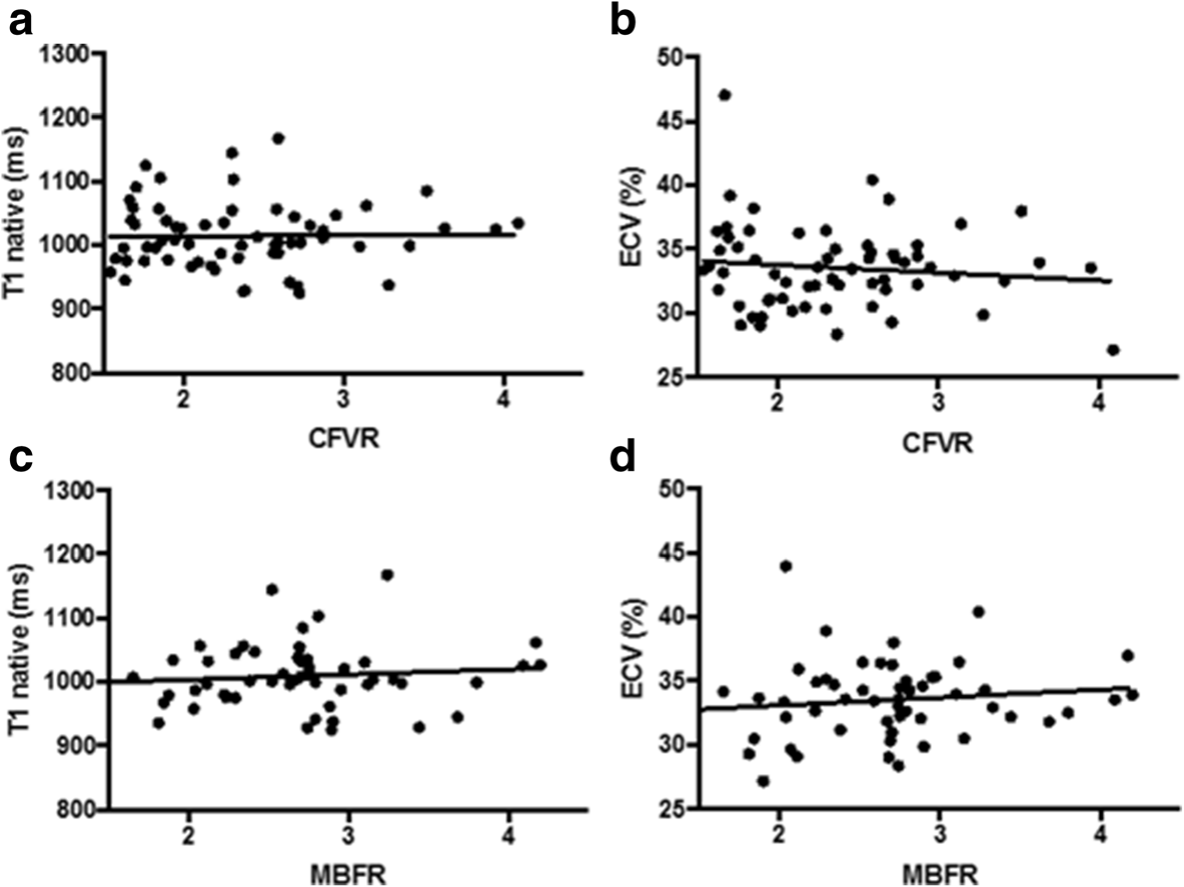
Correlation between measures of coronary microvascular function and diffuse myocardial fibrosis. Legend: **a**. CFVR vs. native T1; **b**. CFVR vs. ECV, **c**. MBFR vs. native T1, **d**. MBFR vs. ECV

No significant correlation was found between CFVR and ECV or native T1, *R* ^*2*^ = 0.02; *p* = 0.27 and *R* ^*2*^ = 0.004; *p* = 0.61 respectively). Similarly, we did not find a correlation between MBFR and ECV or native T1 (*R* ^*2*^ = 0.1; *p* = 0.13 and *R* ^*2*^ = 0.004, *p* = 0.64, respectively) (Fig. 4). For the 23 participants with CFVR below 2 mean (SD) native T1 was 1046 (123) and ECV 34.5 (4.5). For the 19 women with MBFR below 2.5 mean (SD) native T1 was 1031 (134) and ECV was 33.3 (3.8). For those women with TTDE and PET defined CMD there was no difference in ECV and native T1 compared to women with normal CFVR and MBFR, *p* = 0.71 and *p* = 0.36 respectively. Furthermore, there was no correlation between MBFR and ECV or native T1 according to the coronary artery territory (data not shown).

## Discussion

In this study no patient with angina had focal fibrosis and we found no association between the degree of CMD assessed by TTDE and PET and the presence of diffuse myocardial fibrosis measured by CMR, indicating that myocardial ischemia in this population does not elicit myocardial fibrosis. This is a new finding, since no study to date has examined the presence of diffuse myocardial fibrosis in women with angina pectoris and no obstructive CAD.

Arnold et al. investigated 50 patients with diabetes without CAD and 19 matched controls with T1 mapping [40]. There was no difference in left ventricular volume measurements between the two groups, but diabetic patients had significantly shorter post-contrast T1 indicating diffuse myocardial fibrosis. This is interesting since diabetes is a disease characterized by microvascular disease. We excluded women with diabetes to avoid this confounder, but did not find a similar association between CMD and diffuse myocardial fibrosis. The prevalence of other cardiovascular risk factors in our study was relatively high compared to a large Danish normal population of women of similar age [41] but comparable to a large Danish study of 2253 women with angina pectoris and no obstructive CAD [1]. There was however, no clear association between cardiovascular risk factors and presence of diffuse myocardial fibrosis, and HeartScore and Framingham risk scores did not predict more fibrosis. A MESA (multi-ethnic study of atherosclerosis) study also examined the association between T1 mapping values and cardiovascular risk factors in 1208 subjects (49 % women) and found a poor correlation between risk scores and presence of diffuse myocardial fibrosis, particularly in women [42]. These authors concluded that there was a clinical potential for T1 mapping to be used in complement with risk scores to add prognostic value. In another MESA study of 1231 subjects (51 % women) with no focal fibrosis on CMR, women had significantly higher ECV and native T1 compared to men, indicating more fibrosis, and ECV was associated with age in women after multivariable adjustment [43]. This has also been found in another study of 81 healthy controls [44]. We did not see any association with age, but more fibrosis was associated with less hypertension and less atherosclerosis on the CAG, which should be cautiously interpreted due to the small study sample size. The latter could also indicate that the mechanisms causing obstructive CAD, diffuse myocardial fibrosis and CMD in this population are distinct. Both native and post-contrast T1 increased significantly from apex to base, which has also been seen in another study in the unaffected part of the myocardium in patients with myocardial infarction [45].

The cut-off used to define CMD is currently unclear and should be seen as a continuum [9], but most agree that values below 2.0 or 2.5 indicate CMD [3, 46, 47]. In yet unpublished data from the iPOWER study we have, however demonstrated that MBFR is systematically higher than CFVR. We found that 36 % had CMD using a CFVR cut-off value of 2 and 35 % had CMD using a MBFR cut-off value of 2.5. Only 6 (11 %) had MBFR below 2. This prevalence is similar to other studies investigating CMD in this patient population [5, 31, 48, 49]. Impaired CMD was associated with hypertension and higher resting heart rate which is similar to findings from other studies [3, 31, 50, 51].

### Strengths and limitations

This study used multiple imaging modalities to examine the association between CMD and presence of diffuse myocardial fibrosis. The participants were consecutively included and examined systematically and all had a clinical invasive CAG ruling out obstructive CAD. The use of Doppler echocardiography measuring CFVR is a difficult method that requires training and in un-experienced hands this would be a limitation. We assessed repeatability of TTDE CFVR in our group and found an intra class correlation coefficient of 0.97 (0.92;1.00) and coefficient of variation (CI) of 7 % [3, 10] for repeat examinations. The study population was rather small and we cannot rule out that the variation in T1 values and CMD is too small to catch a possible association. However, the study size is fair for an imaging study and the distribution of cardiovascular risk factors was high, indicating that we have included women at risk. Also, the duration of time between the different examinations was relatively large. However, very few subjects had changes in their medication and cardiac symptoms in-between examinations and the level of pharmacological treatment was the same throughout the study. None of the participants went through further clinical evaluation during the study period. This was addressed by having all participants filling out a questionnaire regarding their cardiac symptoms and clinical evaluation, as well as checking the electronic medical chart for new prescriptions during the time interval. Women with no angina but only dyspnea as key symptom leading to a clinical CAG were not included in iPOWER which could potentially induce sampling bias. However, angina is the most common symptom of ischemia and women with only dyspnea and no obstructive CAD are likely to have another explanation to their symptoms than myocardial ischemia.

## Conclusion

In women with angina pectoris and no obstructive CAD we found no association between CMD defined by impaired CFVR or MBFR and diffuse or focal myocardial fibrosis measured by CMR derived T1 or ECV. This indicates that these methods and measurements may provide independent information about different aspects of myocardial and coronary disease in this population.

### Perspectives

That diffuse myocardial fibrosis is not a consequence of CMD in women with angina and no obstructive CAD adds knowledge to the field regarding mechanisms causing angina in these subjects. Such women represent a large patient group in increased risk of cardiovascular events for whom there is currently no effective treatment. Future prospective large-scale studies are required to define the mechanisms causing the diverse symptoms in this population.

## Abbreviations

AHA: American Heart Association
BMI: Body mass index
BP: Blood pressure
CAD: Coronary artery disease
CAG: Coronary angiography
CFV: Coronary flow velocity
CFVR: Coronary flow velocity reserve
CMD: Coronary microvascular dysfunction
CMR: Cardiovascular magnetic resonance
ECG: Electrocardiography
ECV: Extracellular volume
ESC: European Society of Cardiology
HR: Heart rate
IQR: Interquartile range
LAD: Left anterior descending artery
LCX: Left circumflex artery
LVH: Left ventricular hypertrophy
MBF: Myocardial blood flow
MBFR: Myocardial blood flow reserve
PET: Positron emission tomography
RCA: Right coronary artery
SD: Standard deviation
TTDE: Transthoracic Doppler echocardiography
